# An Adaptive TE-PV Hybrid Energy Harvesting System for Self-Powered IoT Sensor Applications

**DOI:** 10.3390/s21082604

**Published:** 2021-04-08

**Authors:** Mahmuda Khatun Mishu, Md. Rokonuzzaman, Jagadeesh Pasupuleti, Mohammad Shakeri, Kazi Sajedur Rahman, Shuza Binzaid, Sieh Kiong Tiong, Nowshad Amin

**Affiliations:** 1 Institute of Sustainable Energy (ISE), Universiti Tenaga Nasional, Kajang 43000, Selangor, Malaysia; mahmuda.khatun@uniten.edu.my (M.K.M.); rokonuzzaman@uniten.edu.my (M.R.); mshakeri@uniten.edu.my (M.S.); siehkiong@uniten.edu.my (S.K.T.); 2 College of Engineering (COE), Universiti Tenaga Nasional, Kajang 43000, Selangor, Malaysia; 3 Solar Energy Research Institute, Universiti Kebangsaan Malaysia, Bangi 43600, Selangor, Malaysia; sajed@ukm.edu.my; 4 Smart Microgrid Advanced Research and Technology (SMART) Center, Department of Electrical and Computer Engineering, Prairie View A&M University, Prairie View, TX 77446, USA; shbinzaid@pvamu.edu

**Keywords:** energy harvesting (EH), hybrid energy harvesting (HEH), solar photovoltaic, thermoelectric, internet of things (IoT), wireless sensor networks (WSNs), low power electronic devices

## Abstract

In this paper, an integrated thermoelectric (TE) and photovoltaic (PV) hybrid energy harvesting system (HEHS) is proposed for self-powered internet of thing (IoT)-enabled wireless sensor networks (WSNs). The proposed system can run at a minimum of 0.8 V input voltage under indoor light illumination of at least 50 lux and a minimum temperature difference, ∆*T* = 5 °C. At the lowest illumination and temperature difference, the device can deliver 0.14 W of power. At the highest illumination of 200 lux and ∆*T* = 13 °C, the device can deliver 2.13 W. The developed HEHS can charge a 0.47 F, 5.5 V supercapacitor (SC) up to 4.12 V at the combined input voltage of 3.2 V within 17 s. In the absence of any energy sources, the designed device can back up the complete system for 92 s. The sensors can successfully send 39 data string to the webserver within this time at a two-second data transmission interval. A message queuing telemetry transport (MQTT) based IoT framework with a customised smartphone application ‘MQTT dashboard’ is developed and integrated with an ESP32 Wi-Fi module to transmit, store, and monitor the sensors data over time. This research, therefore, opens up new prospects for self-powered autonomous IoT sensor systems under fluctuating environments and energy harvesting regimes, however, utilising available atmospheric light and thermal energy.

## 1. Introduction

The electronic devices and networks annex (EDNA) reports that by 2022, there will be around 50 billion devices connected to the internet [1]. Some estimates even claim this number could exceed 100 billion [2]. The wave of IoT is emerging very fast and becoming part of the mainstream electronic industry. Thus, people and society tend to adopt smart devices. These devices are equipped with a wireless terminal and effective sensors connected in a network that can gather data, features, statistics, and all sorts of information from the surrounding environment. Internet connections in embedded systems, controllers, transport systems, wearable devices, commercial security systems, and other objects are envisioned. IoT-based devices need an uninterrupted power supply to ensure the operation of their activities [3]. Therefore, providing the necessary power to maintain all the devices operational for their projected lifetimes is challenging. The corresponding energy demand for IoT devices is very considerable due to their limited energy sources, replacement barriers, ecological obstacles, environmental risk, etc. [4,5]. Future projections for WSNs to allow the IoT indicates a doubling between 2018 and 2023, which will result in a substantially higher energy demand [6]. Till now, the most significant energy sources for IoT sensors are batteries. An estimate shows that more than 23 billion batteries will be needed to power up the IoT devices in 2025 [1], but the rising demand for the batteries needed to power up the IoT appliances is harmful because batteries contain harmful chemicals including lead, cadmium, zinc, lithium, and mercury. Using a battery is also challenging in remote areas because of limited charging facilities and accessibility for replacement. Therefore, energy harvesting (EH) from ambient energy sources, such as light, heat, radio frequency (RF), vibration, etc., is inevitable [7,8,9,10]. This would be an efficient solution to overcome the limitations and mitigate the energy demand for uninterrupted functioning by powering up the billions of IoT devices. In this context, numerous EH systems have been developed for outdoor and indoor applications. However, ambient resources provide low power and are dependent on time-varying environmental parameters, which is insufficient to power up IoT sensors sequentially. Based on the power generation capacity, a single energy source is often insufficient to power up all the sensor nodes; therefore, additional energy sources may be introduced as a secondary power supply. The world’s first multiple or hybrid power system comprising PV and diesel power was started up on 16 December 1978, in the Papago Indian Village (Schuchuli, AZ, USA) [11]. Nowadays, hybrid EH systems are increasingly gaining recognition among researchers and industry. Tadesse et al. proposed an electromagnetic energy source (ES) paired with a piezoelectric ES. The fabricated prototype produced 0.25 W using the electromagnetic mechanism and 0.25 mW using the piezoelectric mechanism, at 35 g acceleration and 20 Hz frequency [12]. Guilar et al. proposed an energy-saving photodiode array, which can generate 225 μW/mm^2^ at 20,000 lux [13]. Two energy sources—RF energy and TEG— with 78% efficiency were fabricated by Lhermet et al. [14]. The harvested energy can run 30 integrated chips (ICs) and consumes 5 nW power. Based on the priority, only one source can provide the required power at a time. The main drawback of this proposed system is that it cannot generate power simultaneously. The authors in [15] recommended a PV-TEG dependent HEH system for the indoor ambient environment. Average power of 621 mW is extracted in the integrated HEHS device at an irradiance of 1010 lux, nearly three times as much energy is obtained in single-source EH. Authors in [16] proposed a modular design that pulls in its power from each linked harvesting device. Using a lithium-ion (Li-ion) or nickel-metal hydride (NiMH) battery extends the system’s dependability. With the inclusion of three energy sources, PV, piezoelectric (PZT), and RF, the authors in [17] proposed a multi EH device that can provide up to 2.5 V and total power of 6.4 mW. A platform combining three distinct EH sources from PV, TEG, and PZT with the input voltage range of 20 mV–5 V is proposed in [18]. A time-based power monitoring system is used to track the harvesters’ power, and a peak efficiency of 96% is achieved whereas, the inductor sharing for the PV boost performance is 78%, TEG boost is 86%, and PZT is 83%. In [19], an MPPT EH device with an expandable control for charging and discharging a lithium polymer (LiPo) battery is proposed for PV and vibration energy. The device shows an overall efficiency of 75–85% for 24-h experiments in a WSN. A battery-free energy harvester based on thermal and the vibration energy is designed in [20] for aircraft health monitoring. The use of a low bias current of only 10 nA per branch ensures low power consumption. Dini et al. [21] designed an autonomous, self-starting, battery-less energy harvester for wearable devices and WSN combining PZT, PV, and TEG transducers. The total current consumption is 47.9 nA per source during all the energy sources are enabled. The test shows the peak single-source efficiency is 89.6%. G. Chowdary et al. [22] presented a HEH device with available power levels of 25 nW–100 µW. The 180 nm chip has an output voltage of 1.5 V with the highest efficiency of 87%. Table 1 summarises a comparison of different hybrid energy harvesting systems. 

In this study, an ambient source-based hybrid energy harvester (HEH) is developed to power the IoT-enabled WSNs continuously. A small solar PV cell and thermoelectric generator (TEG) are used to develop the HEH device. Among the two sources, light sources are abundant in the environment. The PV cell can work well in an indoor or outdoor location. Thus, it will work as the primary source of the proposed system, and the TEG will work as the secondary ES. An ESP32 Wi-Fi module connects the complete system with the internet to monitor the sensor data. For energy backup, an SC is used. The proposed HEHS can overcome the limitations of a single-source energy harvester. It will mitigate the IoT sector’s energy demand, extend the sensor life span and the integrated system.

The rest of this paper is organised as follows: The EH methodology for WSNs is introduced in Section 2, which is divided into three parts: ambient energy sources in Section 2.1, solar energy harvesting system in Section 2.2, and thermal energy harvesting system in Section 2.3. After introducing the EH sources, Section 3 presents the proposed HEHS. Section 4 describes the experimental setup and methodology, including the simulation model, hardware components and the complete prototype. In Section 5, the simulated and experimental results are presented and discussed. Finally, conclusions are drawn in Section 6.

## 2. EH System for WSNs

Recently, WSNs have gained a lot of interest owing to their ubiquitous existence and extensive application in the IoT era. However, a significant bottleneck in WSN technology is the minimal energy associated with WSNs. The design and production of robust and high-performance EH systems for WSN environments are being studied to address this significant constraint. In this section, a brief taxonomy of two different energy sources are discussed. 

### 2.1. Ambient Energy Sources

Outdoor environments have different features and functionality than indoor environments. An abundance of artificial energy sources in indoors are workplaces, banks, clinics, restaurants, and warehouses, etc., [4]. The typical indoor energy sources are presented in Table 2. The most common indoor energy sources are classified into four categories: ambient (A), irregular (I), continuous (C), predictable (P) and partially predictable (PP). 

Table 3 shows the power densities of various indoor energy sources and the technologies per unit length. Under indoor illumination, artificial lighting sources seem pretty dim. In the indoor environment, with the high irradiance, a solar cells power density is 0.1 mW/cm^2^, relative to 100 mW/cm^2^ in outdoor normal monitoring conditions. All artificial energy sources total capacity is smaller than outdoor energy sources by a margin of 10 µW-100 mW. To work in the indoor environment, WSNs need to increase their energy sources over their lifetime. Most of the experiments with vibration or piezoelectric energy, electromagnetic or RF energy, demonstrated the limitations in instantaneous power generation compared to solar and thermal energy. However, currently research is shifting gradually towards piezoelectric and RF energy to overcome the challenges in power generations. On the other hand, the published literature shows that solar and thermal energy sources share identical power densities among all low-power indoor/outdoor energy sources. Therefore, based on the availability and ease of installation, the authors have chosen to utilise solar and thermal energy sources to design the proposed HEH device. The proposed system can increase the performance of the WSNs in an indoor/outdoor climate. WSNs will use thermal energy to remain driven while solar energy is not available. Another key goal of the proposed HEHS is to harvest simultaneous energy when both energy sources are available [1,23].

### 2.2. Solar Energy Harvesting System

**Figure 1:**
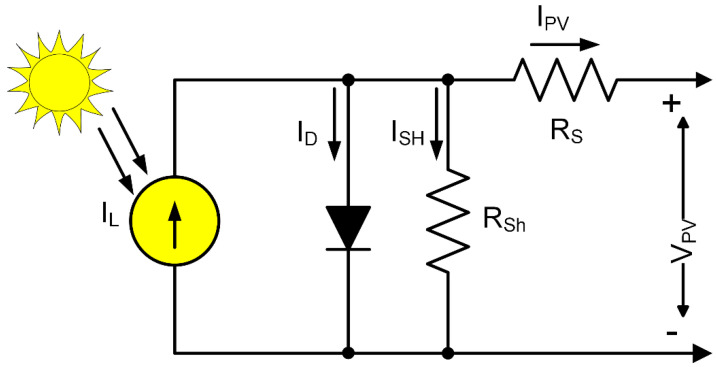
The equivalent electrical circuit of a single diode solar PV cell [30,34].

International Renewable Energy Agency (IRENA) reported that solar energy is one of the most common GreenTech energy sources in 2018 [26]. Naturally, light energy sources are ample, cheap and produce the maximum power density of 10 mW/cm^2^ to 100 mW/cm^2^ on a sunny day. That renders solar PV energy a promising energy source to grow IoT sensor applications [4,25,27,28,29]. Various mathematical models have been discovered to illustrate how solar cells work [30,31,32,33]. This paper considers the single diode electrical circuit model to be the analogous photovoltaic type, as shown in Figure 1.

As light strikes a p-n junction layer, charges are formed, then transmitted through the system to generate electricity. Let, $I_{sc}$ is the short circuit current, $I_{o}$ is the saturation current, a is the ideality factor of the diode, $N_{s}$ is the number of cells is series-connected, T is the PN junction temperature, $K=1.38\times 10^{-23}\;J/K$ is the Boltzman constant, $q=1.6\times 10^{-19}\;C$ is the electron charge, $R_{S}$ is the series resistance, and $R_{sh}$ is the shunt resistance. The output current of the solar cell, $I_{PV}$ can be expressed as:

(1) $$I_{PV}=I_{SC}-I_{o}\left[exp\left(\frac{V_{PV}+R_{S}I_{PV}}{\frac{N_{s}KT}{q}a}\right)-1\right]-\left(\frac{V_{PV}+R_{S}I_{PV}}{R_{sh}}\right)$$

Let, the thermal voltage of the PV cell is, $V_{t}=\frac{KT}{q}$, and $V_{R_{S}}=R_{S}I_{PV}$, Equation (1) can be rewritten as:

(2) $$I_{PV}=I_{SC}-I_{o}\left[exp\left(\frac{V_{PV}+V_{R_{S}}}{N_{s}V_{t}}\right)-1\right]-\left(\frac{V_{PV}+R_{S}I_{PV}}{R_{sh}}\right)$$

The power of the solar PV module can be determined as:

(3) $$P_{PV}=V_{PV}\times I_{PV}\;=V_{PV}\times \left[I_{SC}-I_{o}\left\{exp\left(\frac{V_{PV}+V_{R_{S}}}{N_{s}V_{t}}\right)-1\right\}\right]\;=\;V_{PV}I_{SC}-V_{PV}I_{o}\left[exp\left(\frac{V_{PV}}{N_{s}V_{t}}\right)-1\right]\;=V_{PV}I_{SC}-V_{PV}I_{o}\left[exp\left(\frac{V_{PV}}{N_{s}V_{t}}\right)\right];\;\;\;\;\;\;\;\;\;Since,\;exp\left(\frac{V_{PV}}{N_{s}V_{t}}\right)\gg 1$$

The voltage drop across the series resistance, $V_{R_{S}}=R_{S}I_{PV}$ can be neglected since the value of $V_{R_{S}}$ is even smaller than the PV output voltage. The light illumination is G, and the cross-sectional area is A. Thus, the PV cell efficiency can be calculated by Equation (4):

(4) $$\eta =\frac{P_{PV}}{G\times A}\times 100\%$$

### 2.3. Thermal Energy Harvesting System

**Figure 2:**
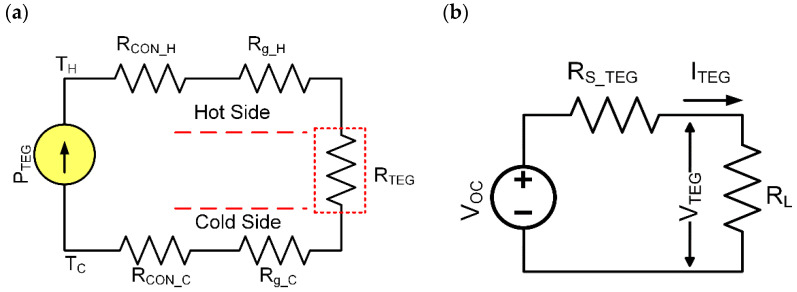
(**a**) Equivalent electrical circuit of TEG; (**b**) simplified circuit of TEG [15].

In 1821, Thomas Johann Seebeck discovered an electric current could exist between two wires separated by a small distance. In honor of the inventor, this effect is formally known as the ‘Seebeck effect’ [24,35]. A Seebeck effect module or thermoelectric generator (TEG) is used in the thermal EH system, transforming the thermal energy into electric energy [35,36,37]. The thermal energy produced from the heat source at a certain high temperature *T_H_* (hot side temperature). *T_H_* is channelled through the enclosed TEG through a thin film of thermal and electrically conductive silver grease between them to the heat sink. The excess heat stored in the heat sink is then emitted to the local ambient air at a lower *T_C_* (cold side temperature) temperature. An analogous electrical circuit model of the TEG is given in Figure 2.

Figure 2a shows the temperature gradient, ∆*T* (∆*T* = *T_H_ − T_C_*) is higher than the temperature difference, ∆*T_TEG_*. The thermal contacts resistance for the hot side (*R_CON_H_*) and cold side (*R_CON_C_*), as well as the thermal grease resistances for hot side (*R_g_H_*) and cold side (*R_g_C_*), exist in the thermal energy harvester (TEH); therefore, the temperature difference is externally imposed across the junction point of TEG. The thermal resistance, *R_TEG_* of the TEG module, is made to be as maximum as possible to reduce this negative effect, and the rest of the TEH resistance is maintained as low as possible. By considering these design factors, the TEH, with a 30 mm × 30 mm × 3.8 mm physical size, is selected to channel the bulk of the thermal heat through TEG to maximize TEH. To determine the TEG’s efficiency in powering, the IoT enabled sensor applications, study and characterization work was carried out on the assembled TEH. Seebeck’s effect indicates that the open-circuit voltage, *V_OC_*, of the TEG enclosed in the TEH, composed of ‘n’ number of thermocouples connected electrically in series and thermally in parallel. Therefore, *V_OC_* can be formulated in Equation (5):

(5) $$V_{OC}=S\times \Delta T\;=n\times \alpha \left(T_{H}-T_{C}\right)$$

where *α* = Seebeck’s coefficient of a thermocouple and *S* = Seebeck’s coefficient of a TEG. Figure 2a can be simplified as Figure 2b. According to the applied temperature difference, ∆*T*, an electrical current, *I_TEG_*, flows to *R_L_* load resistance. Let *R_S_TEG_* is the internal resistance of the TEG, then the electrical current through the TEG can be determined as:

(6) $$I_{TEG}=\frac{V_{OC}-V_{TEG}}{R_{S\_TEG}}\;=\frac{\left[n\times \alpha \left(T_{H}-T_{C}\right)\right]-V_{TEG}}{R_{S\_TEG}}$$

Now, the harvested power from the TEG, *P_TEG_* can be expressed by the equation below:

(7) $$P_{TEG}=V_{TEG}\times I_{TEG}\;=V_{TEG}\times \left[\frac{\left[n\times \alpha \left(T_{H}-T_{C}\right)\right]-V_{TEG}}{R_{S\_TEG}}\right]$$

## 3. Proposed Hybrid Energy Harvesting System (HEHS)

**Figure 3:**
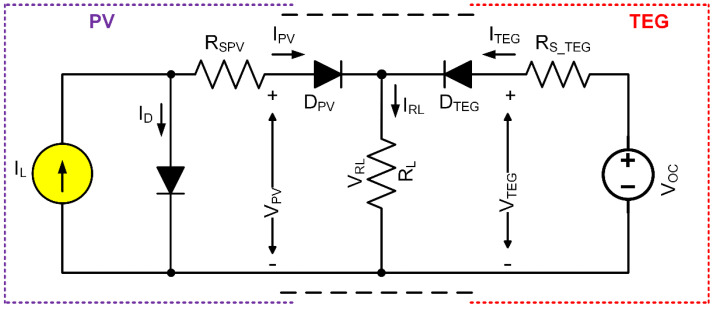
The equivalent electrical circuit of the proposed HEHS.

HEHS contains two or more energy sources that may potentially include solar PV, thermal, wind, piezoelectric-vibration, geothermal, hydropower, biomass, natural gas, oil, coal, or nuclear energy [38,39,40,41,42,43]. The idea of HEHS has recently been explored in the literature as a possible micro-power supply solution to reduce the size of the energy supply and prolong the working life of the IoT enabled sensor applications. Researchers also introduced a variety of HEHS to integrate numerous small-scale EH sources [44]. For these approaches, each energy harvesting source needs a unique power control circuit to change the power flow from the energy source to its output load or the sensor [45]. The concern is that multiple of the expanded energy sources must raise the number of converters used. Thus, the proposed HEHS needs only one single-power electronic converter with a single low-power control circuit to simultaneously condition solar and thermal energies’ combined performance. By eliminating the usage of separate power storage units for various energy sources, the number of components used in the HEH device is decreased, and the shape, cost and power losses of the system are minimized. However, the proposed approach may have an impedance mismatch between interconnected energy sources. The equivalent electrical circuit of the proposed HEHS is shown in Figure 3. 

To block the inverted current flow, the solar PV modules output voltage, *V_PV_* is connected to the load, *V_RL_* via a Schottky diode, *D_PV_*. Similarly, the TEG modules output voltage, *V_TEG_* is directly connected to the load, *V_RL_* via another Schottky diode, *D_TEG_*. The technical specifications of the two energy sources show that the output voltages are not too low. If the proposed hybrid systems energy sources are connected in series, then the total voltage will be increased. Thus, the energy sources are configured with a parallel connection that enhances more current flow. From Figure 3, the load voltage and the load current for the energy sources can be represented as:

(8) $$V_{RL}=V_{PV}+D_{PV}=V_{TEG}+D_{TEG}$$

(9) $$I_{RL}=I_{PV}+I_{TEG}$$

where *V_PV_* = load voltage, *D_PV_* = diode voltage, *I_PV_* = load current of the solar PV module, respectively. *V_TEG_* = load voltage, *D_TEG_* = diode voltage, *I_TEG_* = load current of the TEG, respectively. *V_RL_* and *I_RL_* are the hybrid load voltage and current, respectively. The diodes, *D_PV_* and *D_TEG_* consume 0.2 V, each as the forward voltage drop. The solar PV power from Equation (3) and TEG power from Equation (7) is combined. Then, the diodes power losses are subtracted. Hence, the hybrid electrical power, *P_HEH_* throughout the HEH can be expressed as:

(10) $$P_{HEH}=P_{PV}+P_{TEG}\;=V_{PV}I_{SC}-V_{PV}I_{o}\left[exp\left(\frac{V_{PV}}{N_{s}V_{t}}\right)\right]+V_{TEG}\times \left[\frac{\left[n\times \alpha \left(T_{H}-T_{C}\right)\right]-V_{TEG}}{R_{S_{TEG}}}\right]\;=V_{RL}I_{SC}-V_{RL}I_{o}\left[exp\left(\frac{V_{RL}}{N_{s}V_{t}}\right)\right]+V_{RL}\times \left[\frac{\left[n\times \alpha \left(T_{H}-T_{C}\right)\right]-V_{RL}}{R_{S\_TEG}}\right]$$

## 4. Experimental Setup and Methodology 

**Figure 4:**
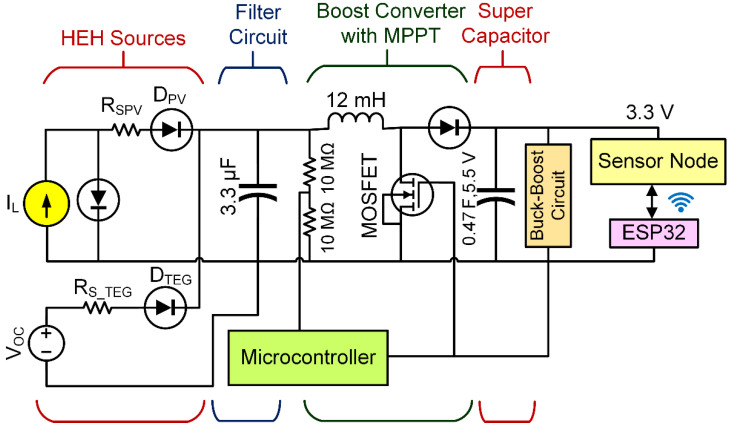
Simulink functional model of the proposed HEHS.

Both simulation and hardware trial are effectively performed to verify the proposed HEHS. MATLAB/SIMULINK 2020b platform is used for the simulation. Figure 4 shows the proposed SIMULINK model with functional blocks of HEHS. The proposed model includes the energy sources, filter circuit, boost converter with maximum power point tracking (MPPT) technique [46,47,48]. The MPPT unit controls the pulse width modulation (PWM) and operates the energy sources near the maximum power point (MPP). 

**Figure 5:**
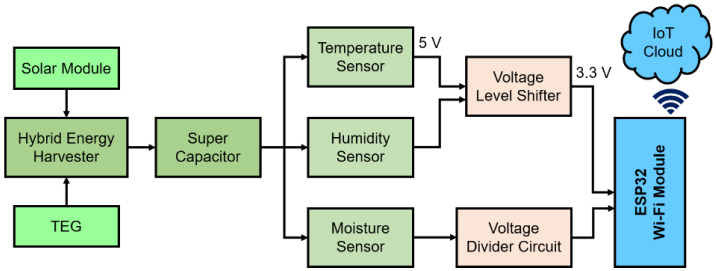
Functional block diagram of the proposed HEH prototype.

Solar and thermal energy sources are nonlinear; thus, energy storage can perform as a supplementary power source at a steady level. In this work, a 0.47 F, 5.5 V SC is used on the grounds of supremacy over the conventional battery, with more than half a million charging cycles, 10–20 years of service life, high power capacity, etc. [6,25]. A buck-boost converter circuit is used to deliver 3.3 V and 5.0 V to the IoT connected sensors. The ESP32 Wi-Fi module operates at 3.3 V, and the sensors need 5.0 V to work. Figure 5 shows the functional block diagram schematic of the proposed HEH prototype. A solar PV module and TEG are connected parallelly to the hybrid energy harvester module ADP5091. The parallel connection ensures the maximum power transfer from the energy sources to the energy storage and the load. Since the occupied energy sources have few voltage ratings that are not very low, they are not connected in series. The series connection will cause to increase in input voltage. As the system is for a low power energy harvesting system, the input voltage should be kept at a limit. 

### Prototype Design

**Figure 6:**
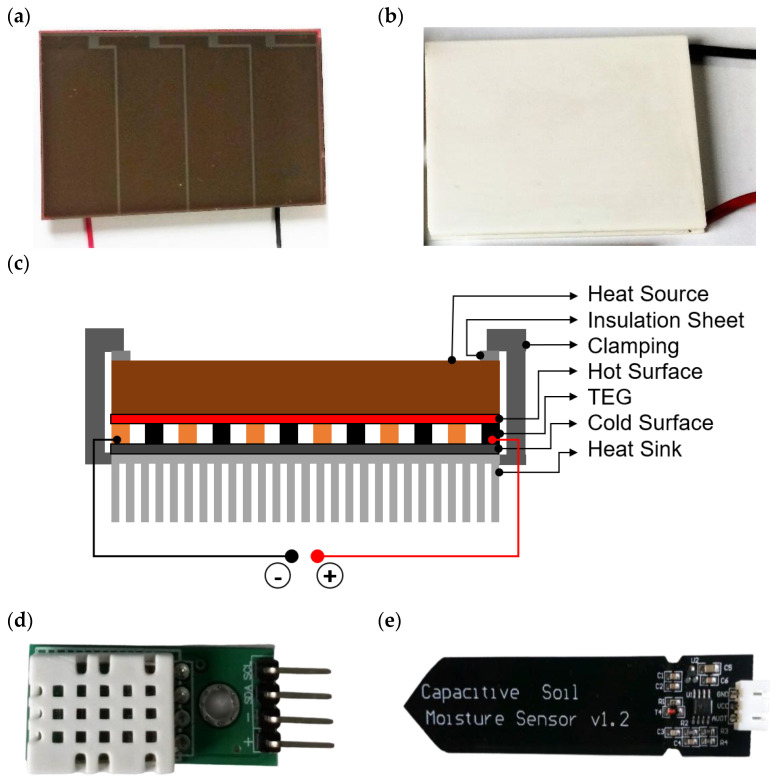
(**a**) AM-1454 solar PV module; (**b**) GM250-71-14-16 TEG module; (**c**) schematic structure of TEG module; (**d**) SHTC3 temperature and humidity sensor; (**e**) SEN0193 V1.2 capacitive soil moisture sensor.

In this research, the integrated amorphous silicon solar cells from Panasonic Solar Amorton Co., Ltd., Tokyo, Japan is used. Figure 6a shows the AM-1454 solar PV module. Table 4 tabulates the technical characteristics of the module which shows the glass type module with four cells connected in series. The dimension of the module is 41.6 × 26.3 × 1.1 mm (width × length × thickness) with 3.0 g weight. The electrical properties are also mentioned under fluorescent light having a luminous intensity of 200 lux and temperature of 25 °C. Figure 6b shows the TEG module of model number GM250-71-14-16, from European Thermodynamics Ltd. (Leicestershire, UK) [49]. The schematic structure of the TEG module is illustrated in Figure 6c. A heating source is located on top of the module, then heated to the p-n junction. The bottom side of the module is covered with a cold surface and a heat sink. Besides the strict feature of low-carbon emission, compact structure, reliable performance, maintenance-free and noise-free operation of this TEG module, the technical specifications are presented in Table 5. This module can generate a maximum of 5.3 V with a maximum temperature of 250 °C and start functioning at 25 °C. Three sensors, namely a moisture sensor (SEN0193), temperature and humidity sensors (SHTC3), are used to design the wireless sensor network. SHTC3 uses proprietary digital-signal-collecting-technique and humidity sensor technologies to ensure its efficiency and stability. The sensor’s output is digitally calibrated, and the sensing components are attached to a single-chip 8-bit device. The sensor in this model is temperature-compensated and measured in an effective calibration chamber. The calibration-coefficient is stored in the one-time programming (OTP) memory software form, and when the sensor is sensed, the coefficient of memory is used. Small size and low consumption and long transmission distance (20 m) make the SHTC3 suitable for all kinds of harsh applications. Single row lined with four sticks, rendering the connection very easy. Figure 6d shows the SHTC3 sensor, which has four leg pins, including power supply (V_DD_) pin, signal pin, null pin and ground (GND) pin. A capacitive moisture sensor having the model number SEN0193 is used. This sensor tests soil moisture levels by way of capacitive sensing rather than resistive sensing. It is constructed of a material that is immune to rust, granting it an exceptional service period. An on-board voltage regulator is included in this module, giving it a working voltage range of 3.3 V~5.5 V. Figure 6e shows the capacitive soil sensor of version 1.2 (v1.2). An intelligent integrated low-power energy management unit, ADP5091 from Analog Devices (Norwood, MA, USA), is used to transform hybrid DC power from solar PV and TEG modules. This energy harvesting module charges the supercapacitor and distributes the harnessed energy to the sensor nodes as required [50].

**Figure 7:**
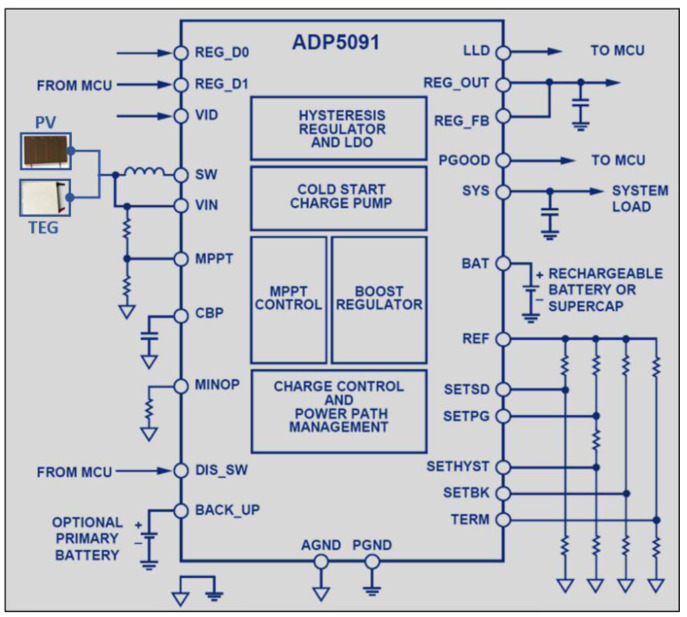
Typical application circuit of ADP5091 EH Module [50].

Figure 7 shows the typical application circuit of the ADP5091 module that enables effective conversion of the harvested minimal power from a range of 6 µW to 600 mW with sub microwatt operational losses. The control unit starts to operate from 380 mV input voltage with the internal cold starting circuit. After the cold start, the regulator runs at an input voltage level from 0.08 V to 3.3 V. An external resistor divider or VID pin may be used to set a control output of an extra 150 mA. The MPPT regulation maintains the input voltage ripple within a defined range to ensure a reliable dc-to-dc boost conversion. Dynamic sensing mode and no sensing mode, all programming control points of the input voltage, enable the most energy to be extracted from the harvester. The programmable minimum operational threshold allows shutdown to be boosted under low input conditions. The Low Light Density (LLD) pin of the ADP5091 is the Minimum Operating Power (MINOP) comparator output as a low light indicator for a microprocessor. The optional primary cell battery can be attached and operated by an integrated power path control block, configured to transfer the power supply from the energy harvester, the rechargeable battery, and the primary cell battery. The ADP5091 is available in a 24-lead Lead Frame Chip Scale Package (LFCSP) and is classified at −40 °C to +125 °C temperature range. 

**Figure 8:**
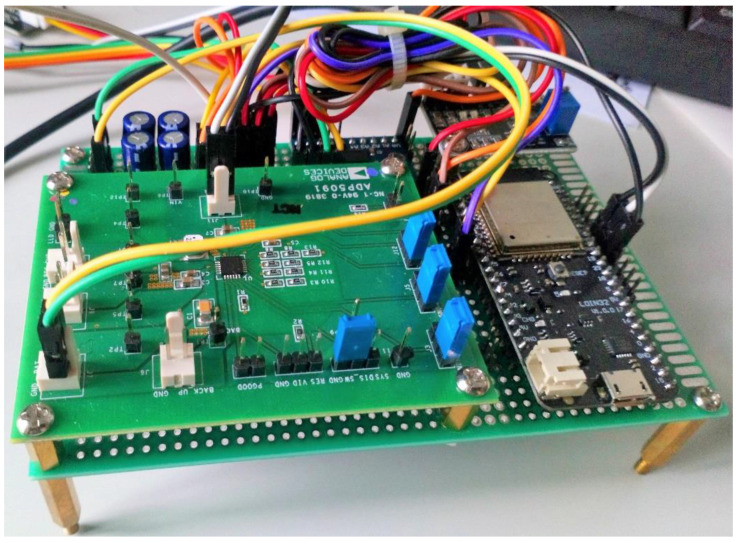
The complete prototype of TE-PV HEH device.

In the prototype, an electric double-layer capacitor (EDLC), part number DDL474S0HF1ERR, 0.47 F, 5.5 V, supercapacitor (SC), is used. A high-speed 8-Bit bidirectional voltage-level shifter module is used to match the varying logic level. Such as 5 V from sensor output/Input port and peripheral modules of 3.3 V. The voltage level translation rate is 2 Mbps (open-drain driving), 60 Mbps (push-pull driving @VCCA = 3.3V), VCCA voltage is 1.2 V–3.6 V, VCCB voltage is 1.65 V–5.5 V and VCCA < VCCB. A voltage divider circuit is used to reduce the output voltage, 5 V of the moisture level sensor, to 3.3 V as the input of the ESP32 module. ESP32, a hybrid Wi-Fi and Bluetooth module from Espressif Systems (Shanghai) Co. Ltd., Shanghai, China, made this study more attractive to implement the wireless sensor network [51]. This modules power usage is remarkably low relative to conventional technologies. The module can operate well in industrial conditions, with temperatures ranging from −40 °C to + 125 °C. ESP32 provides flexibility and durability with minimum PCB specifications. This chip can operate as a complete stand-alone machine or as a slave computer to a host microcontroller unit (MCU), reducing the main application processors overhead. ESP32′s SPI/SDIO or I2C/UART interfaces can communicate with other devices and provide Wi-Fi and Bluetooth capabilities. The complete HEHS prototype with all the components is shown in Figure 8.

**Figure 9:**
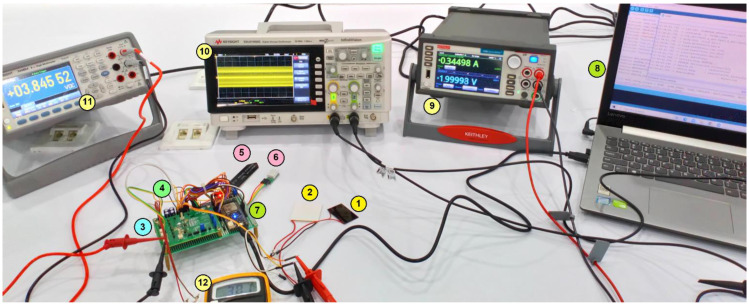
Experimental test bench: (1) AM-1454 solar PV module; (2) GM250-71-14-16 TEG module; (3) ADP5091 EH module; (4) Supercapacitor (0.47 F, 5.5 V); (5) SEN0193 V1.2 capacitive soil moisture sensor; (6) SHTC3 temperature and humidity sensor; (7) ESP32 Wi-Fi module; (8) IoT connection through serial monitor; (9) input voltage; (10) Oscilloscope view of input voltage; (11) charging voltage; (12) SC voltage.

The experimental set up with the connection between all the components is shown in Figure 9. Here, the energy sources in (1) and (2), energy harvester module in (3), SC in (4), sensors in (5) and (6), Wi-Fi module in (7), IoT data communication in (8) are shown, respectively. A 2460 source meter (KEITHLEY, Cleveland, Ohio, USA) shown in (9) is used to measure the input voltage, current and power. The EDUX1002G digital storage oscilloscope (Keysight, Santa Rosa, CA, USA) shown in (10) is used to measure and observe the output signals. A Keysight 34465A 6 ½ digit multimeter shown in (11) is used to measure the charging voltage. A digital multimeter shown in (12) is used to measure the supercapacitor output voltage. 

## 5. Results and Discussion

**Figure 10:**
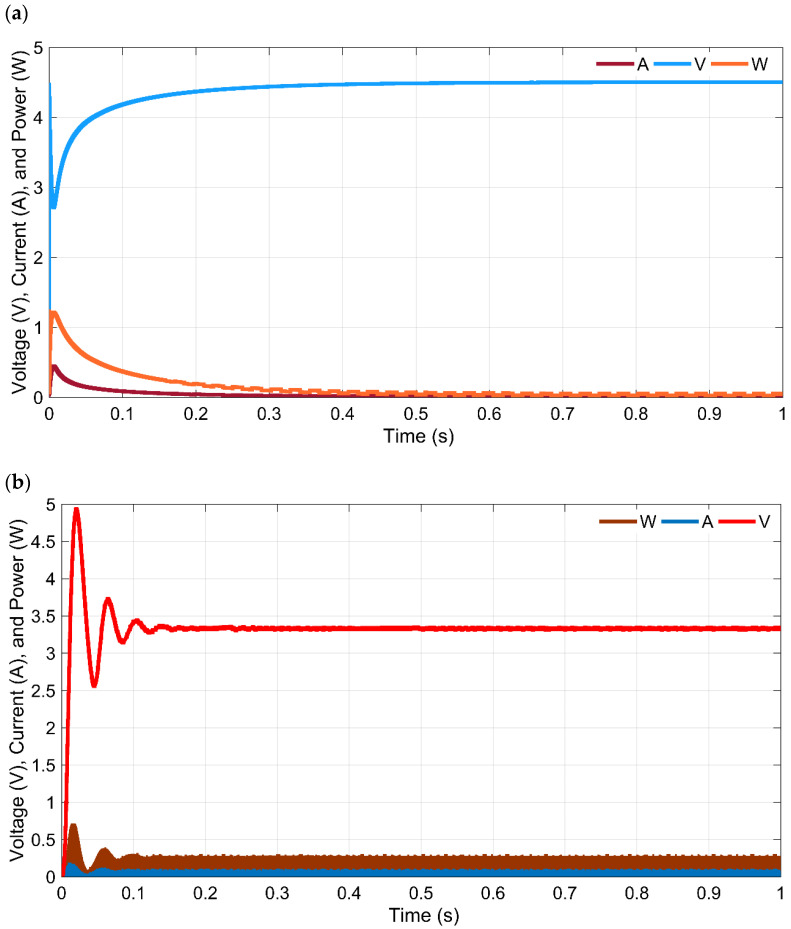

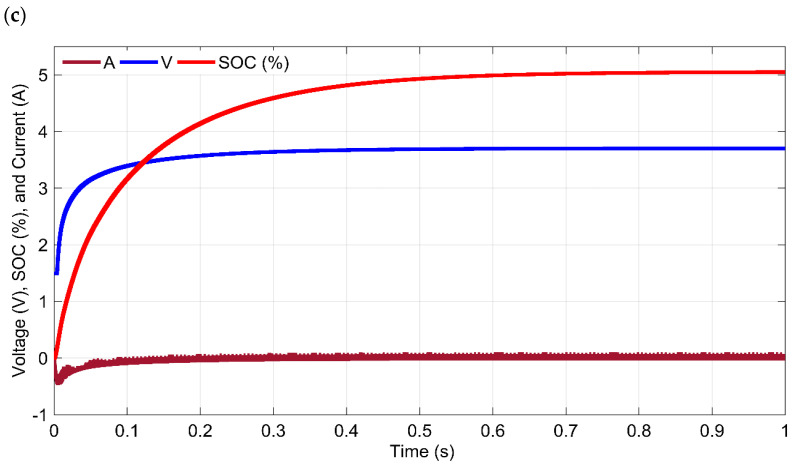
Simulation results (**a**) hybrid output curve of the proposed system; (**b**) boost converter output curve; (**c**) the SC output voltage, current and SOC (%).

A complete prototype of an ambient source-based HEH device is developed to operate a few IoT-enabled sensors. The prototype is tested in the laboratory, and it showed the desired result. Before the hardware implementation, the HEHS is designed and tested in SIMULINK 2020b. Figure 10a shows the simulated output curve of the HEH circuit. The generated voltage raises to 4.5 V maximum at 0.4 s time. Whereas, in the beginning, the hybrid systems output power was 1.2 W and later, it provides constant power of around 0.5 W. Figure 10b shows the boost converter output voltage. Before delivering a constant 3.3 V to the SC, the charging voltage is fluctuating until 0.2 s. From 0.2 s, the converter provides a constant 3.3 V charging voltage. Figure 10c shows the SC voltage, current and state of charge (SOC%) characteristics. SOC of the SC reaches 100% within 0.6 s; the desired output voltage level is 3.3 V, which can accomplish within 0.1 s. Later, SC provides a constant 3.3 V continuously until the energy sources are available. The simulation shows that the SC takes only 0.6 s time to full charge. In this study, only energy sources and energy harvester part are simulated and tested. 

**Figure 11:**
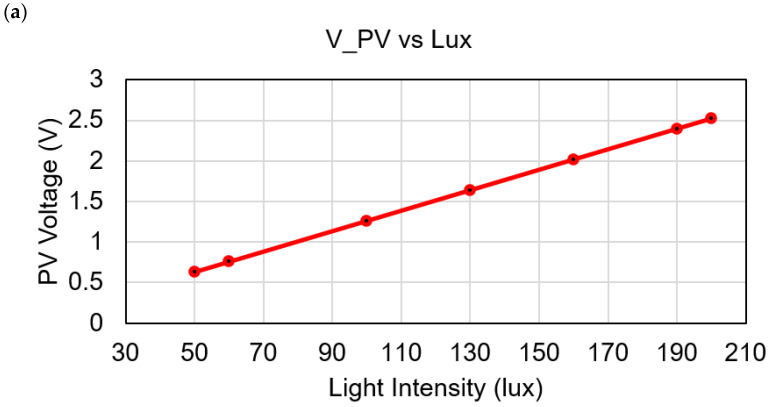

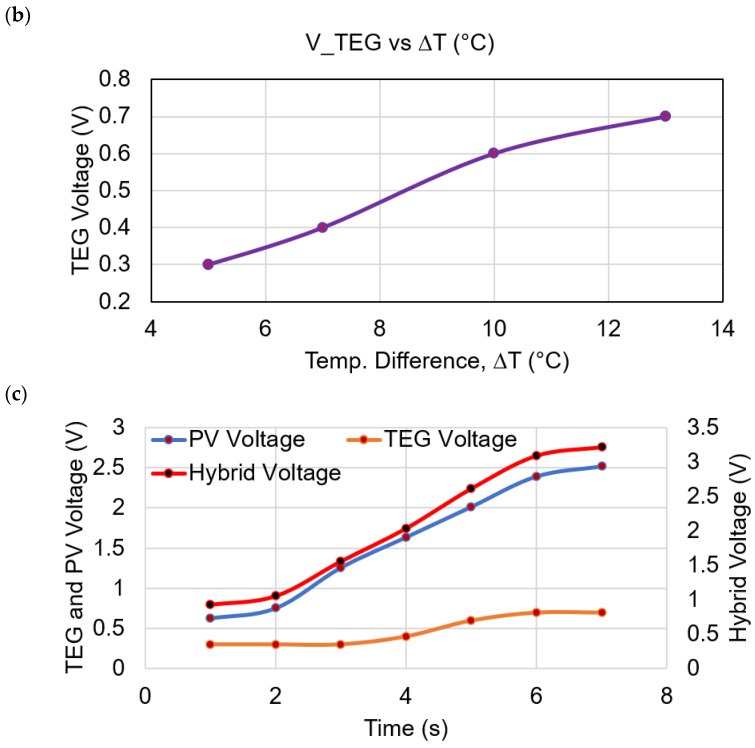
Generated voltages at different light intensity and temperature difference, (**a**) PV voltage vs. lux; (**b**) TEG voltage vs. temperature difference; (**c**) hybrid voltages from PV and TEG.

In the prototype, both solar cell and TEG are connected to the *V_in_* (J11 pin) of the ADP5091 HEH module. *V_in_* is internally connected to the cold start charge pump and the maximum power point tracking (MPPT) pin. The MPPT pin’s output is connected to the boost controller, and the boosted voltage delivered to the BAT (J7 pin) of the HEH device. The BAT pin is directly connected to the supercapacitor (SC). The minimum and maximum input voltage of the ADP5091 is 0.8 V and 3.3 V, respectively. From the experiment, it is shown that the SC starts to charge when the energy sources generate only 0.8 V. In the investigation, the different input voltage level of 0.8 V to 3.2 V is tested. Figure 11a shows the PV cell generated voltage at different light intensity. In indoor environment, at minimum 50 lux of a LED lamp the PV cell can generate 0.63 V and at maximum 200 lux the PV cell can generate 2.5 V. Figure 11b shows the TEG voltage at different temperature. At minimum ∆*T* = 5 °C the TEG can generate 0.3 V and at ∆*T* = 13 °C the TEG generates 0.7 V. The value of ∆*T* is determining by, ∆*T* = *T_H_*-27 °C, where the cold side temperature of TEG is similar as the standard room temperature 27 °C. Figure 11c shows the combined voltages from the TEG and PV.

**Figure 12:**
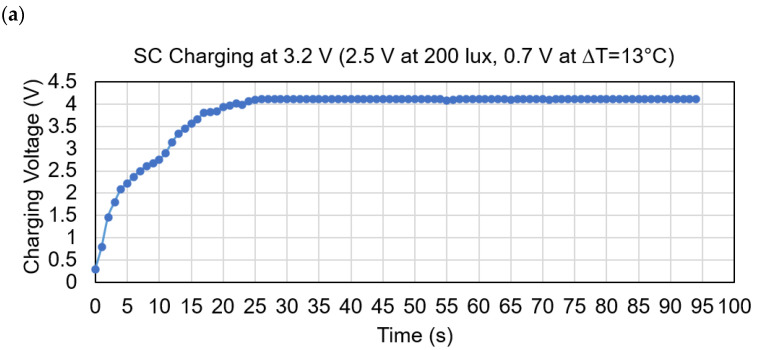

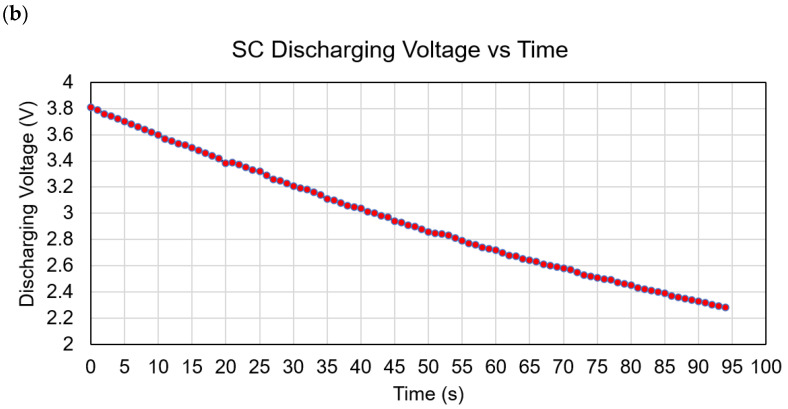
State of charge and discharge, (**a**) Supercapacitor charging at maximum 3.2 V input voltage; (**b**) SC discharging voltage versus time.

At the value of ∆*T* = 13 °C the TEG generates 0.7 V and at the maximum light intensity of 200 lux the PV module generates 2.5 V. In this combination the proposed system produces maximum 3.2 V. The complete charging state of the SC at a maximum of 3.2 V is shown in Figure 12a. It is found that whatever the charging time is, the discharging time is almost the same. Figure 12b shows the SC discharging voltage levels, starting from the full charged level 3.81 V to discharge level 2.28 V per 5 s interval. The average discharging time is recorded 92 s. Hence, the sensors and the IoT function can operate for 92 s uninterruptedly without any energy sources.

**Figure 13:**
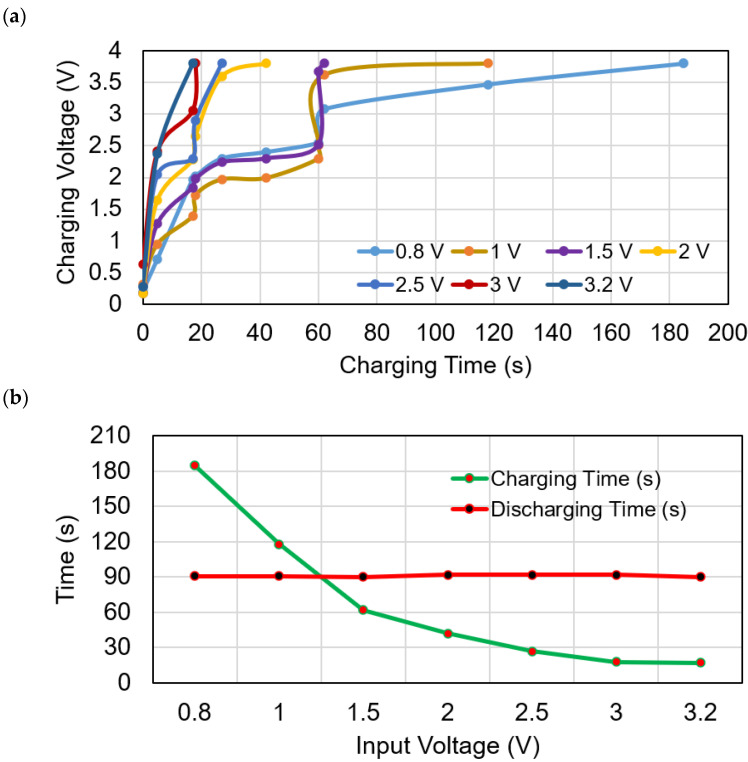
(**a**) charging voltages and the respective times in different input voltages; (**b**) SC charging and discharging voltage versus time.

Figure 13a shows the SC charging voltage at different voltage level available from the energy sources. Since solar power and thermal power are depends on light irradiance and thermal gradient, respectively. Thus, the generated power from both the energy sources are changes linearly based on the fluctuating irradiance and heat difference, respectively. In the indoor environment, both sources generate low power. The combined voltages available from solar and TEG has been taken and divided into different voltage level as 0.8 V, 1 V, 1.5 V, 2 V, 2.5 V, 3 V and 3.2 V. The voltage range 0.8 V–3.2 V is selected, based on the operating voltage limits of the ADP5091. The generated power is stored in the SC through the ADP5091 energy harvester. The SC delivers the required power to the IoT connected sensors. It is observed that the proposed system takes the less charging time of only 17 s at the maximum input voltage of 3.2 V. A maximum of 185 s is needed to charge the SC at the minimum voltage level of 0.8 V. For measurement purposes, 3.8 V has been chosen as the maximum charging voltage level of the SC. In the proposed device, in different input voltages as 1 V, 1.5 V, 2 V, 2.5 V and 3 V, it takes 118 s, 62 s, 42 s, 27 s and 18 s, respectively. Thus, the low input voltage takes a higher time to be charged from the experiment, and the high input voltage consumed less charging time. Figure 13b shows the maximum charging time versus discharging time per specific input voltage. The sensors send data to the IoT server until the SC voltage level is 2.28 V. Below the charge level of 2.28 V, the SHTC3 sensor failed to connect to the server. The used SC takes 230 s time to discharge from 3.81 V to 1 V. It is observed that, at only 1.97 V~2.0 V, the moisture sensor started to operate with data transmission. Still, the SHTC3 sensor started to operate; however, the power is still insufficient to transmit the data. At the voltage of 2.28 V, SHTC3 transmit data to the internet server through the ESP32 Wi-Fi module. Thus, during the discharge, the SC can power up the SHTC3 and SEN0193 sensors until 92 s (operates at 2.28 V minimum) and 120 s (operates at 1.97 V minimum), respectively. 

The temperature and humidity sensor SHTC3 consume 3.77 V, 0.005 mA and 18.85 µW (0.00001885 W) power to run the sensor. The moisture sensor needs 3.76 V, 5.4 mA and 20,304 µW (0.020304 W). The sensors standby mode and data transmission mode total 20322.85 µW (0.20011885 W), (20.32 mW) power consumption is recorded. The experiment found that data transmission per two-second interval, the SC back up the complete system for 92 s. In this time, sensors can transmit 39 successful data strings to the webserver. Faster or slower sensor data sampling rate can consume more or save power, respectively. Thus, based on the available power or the state of charge of the device, it is also possible to save SC power by regulating the data transfer interval, slower or faster the data sampling rate. Proper resource optimisation depending on the data transfer rate, the sleep/wake-up period of the sensors, can save the power that can be implemented in a future paper. 

**Figure 14:**
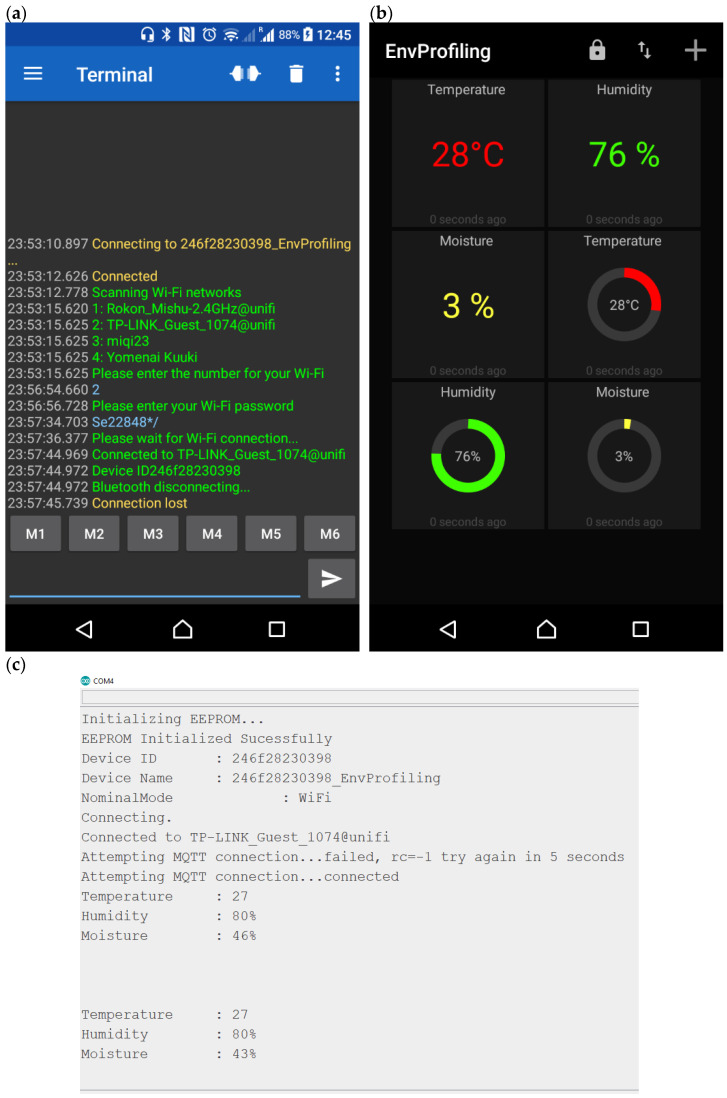
IoT features developed for the HEHS prototype (**a**) connection established to the smartphone; (**b**) MQTT dashboard to monitor sensor data; (**c**) sensor data transmitting to the serial monitor of laptop or cloud server.

The developed IoT framework is functioning perfectly, and different steps are shown in Figure 14a–c. Figure 14a shows the prototype device is interfaced to the smartphone through IoT. If the device finds an available Wi-Fi network, it will be connected to the nearest network; otherwise, it will activate the Bluetooth mode and be connected to the smartphone. Figure 14b shows the live MQTT dashboard updating the sensor data continuously. Figure 14c demonstrates the prototype is linked with the server, and the captured data are displayed on the laptop serial monitor.

## 6. Conclusions

A complete IoT-enabled HEHS has been developed for self-powered WSNs. The proposed design is fully functional in both indoor and outdoor environments. Theoretical studies and the experiments are performed to verify the features of the integrated thermal and solar energy harvesting methods. The prototype HEH device has successfully integrated a low-power HEH power management unit into the hardware prototype. The performance and the sensing, monitoring and data transmission rate are analyzed. The power management circuit with an MPPT-based fixed reference voltage system takes a maximum of 17 s time to fully charge the SC. The SC backup the complete device for 92 s without any energy sources. During this backup time, the sensors transmit 39 data string to the webserver at a two-second interval. The HEH prototype can produce a minimum of 0.14 W at 0.8 V, and 2.13 W at 3.2 V combined input voltage. The device can operate in a low-intensity indoor light illumination of at least 50 lux (0.63 V), a low-temperature difference of TEG at ∆*T* = 5 °C (0.3 V) and high illumination of light at 200 lux (2.5 V) and ∆*T* = 13 °C (0.7 V), respectively. This integrated HEHS is more effective than the traditional single energy harvesting system. Therefore, the developed HEHS has the huge potential to be used for comprehensive environment profiling, smart home applications, remote areas for long term data collection, horticulture applications, etc.

## Figures and Tables

**Table 1:** Comparison of different hybrid energy harvesting systems.

Ref.	Year	Energy Sources	Input Power	MPPT	Storage
[15]	2011	PV, TEG	392 µW	Yes	SC
[16]	2011	PV, Wind	-	Yes	Li-ion
[17]	2011	PV, PZT, RF	7.3 mW	No	None
[18]	2012	PV, TEG, PZT	-	Yes	None
[19]	2014	PV, PZT	PV-60 mW, PZT-3 mW	Yes	SC, LiPo
[20]	2015	TEG, PZT	-	No	None
[21]	2015	PV, TEG, PZT, RF	PV-55 µW, TEG-101 µW, PZT-59 µW	Yes	None
[22]	2016	PV, PZT, RF	20 µW	Yes	None

**Table 2:** Characteristics of indoor energy sources.

Energy Sources	Features	Availability	Observations
Light	A, C, I, P	Indoor/Outdoor	Direct/reflected sunlight, illumination from artificial light
Thermal	A, C, I, P	Indoor	Ambient thermal grading between the machine, the human body
Wind	A, C, P	Indoor/Outdoor	Air circulations from an electric fan or air conditioner
Vibration	A, I, P	Indoor/Outdoor	Human motion, machine vibration
Radio Frequency (RF)	A, I, PP	Indoor	Wi-Fi or mobile network

**Table 3:** The extracted power produced from the familiar ambient sources [24,25].

Energy Source	Harvesting Device	Power Density	Harvested Power
Indoor Light	Solar PV Panel	0.1 mW/cm²	10 µW/cm²
Outdoor Light		100 mW/cm²	10 mW/cm²
Human Thermal	Thermoelectric Generator	20 mW/cm²	30 µW/cm²
Industrial Thermal		100 mW/cm²	1–10 mW/cm²
Human Vibration	Piezoelectric Device Electrostatic	0.5 m at 1 Hz 1 m/s² at 50 Hz	4 µW/cm²
Industrial Vibration	Piezoelectric Electromagnetic	1 m at 5 Hz 10 m/s² at 1 kHz	100 µW/cm²
RF: GSM 900 MHz	Antenna Router	0.3 µW/cm²	0.1 µW/cm²
RF: Wi-Fi		0.015 µW/cm²	0.001 µW/cm²

**Table 4:** Specification of solar PV module AM-1454.

Model No.	Substrate	Fluorescent Light at 200 lux					Dimension W × L × T (mm)	Weight (g)
		V_oc_ (V)	Isc (µA)	V_ope_ (V)	I_ope_ (µA)	No. of Cells		
AM-1454	Glass	2.5	35.2	1.5	33.3	4	41.6 × 26.3 × 1.1	3.0

**Table 5:** Specifications of GM250-71-14-16 TEG module.

Parameter	Value
Hot surface temperature	250 °C
Cold surface temperature	30 °C
Open circuit voltage	5.85 V
Maximum voltage	5.3 V
Maximum current	1.1 A
Output power	2.98 W
Dimension	30 × 30 × 3.8 (mm)

## Data Availability

Not applicable.
